# Higher dialysate calcium concentration is associated with incident myocardial infarction among diabetic patients with low bone turnover: a longitudinal study

**DOI:** 10.1038/s41598-018-28422-w

**Published:** 2018-07-03

**Authors:** Miho Tagawa, Takayuki Hamano, Shinichi Sueta, Satoshi Ogata, Yoshihiko Saito

**Affiliations:** 1 Department of Nephrology, Nara Medical University, Kashihara, Japan; 2 Committee of Renal Data Registry, Japanese Society for Dialysis Therapy, Tokyo, Japan; 3 Center for iPS Cell Research and Application, Kyoto University, Kyoto, Japan; 4 First Department of Internal Medicine, Nara Medical University, Kashihara, Japan

## Abstract

This is a longitudinal study on 53,560 hemodialysis patients from the Japan Renal Data Registry. Predictor was D[Ca] ≥3.0 vs 2.5 mEq/L. Outcomes were the first CV events during 1-year observation period. Association of D[Ca] with CV events and effect modifications were tested using multivariate logistic regression analyses. Diabetes mellitus (DM) was a significant effect modifier for association of higher D[Ca] and myocardial infarction (MI) (OR: 1.26 (1.03–1.55) among DM and 0.86 (0.72–1.03) among non-DM, p for interaction <0.01). The effect size was not affected by further adjustment for serum albumin-corrected Ca or intact parathyroid hormone (iPTH) levels, but was attenuated by adjustment for intradialytic change in serum Ca concentration (ΔCa) (1.16 [0.89–1.51]). Among DM, D[Ca] ≥3.0 mEq/L was significantly associated with MI in the first tertile of corrected Ca or iPTH ≤60 pg/ml (p for interaction 0.03 and 0.03, respectively). In conclusion, higher D[Ca] was associated with incident MI in DM, especially with low serum Ca or iPTH levels. Attenuation of the effect size by adjustment for ΔCa and stratified analyses suggest that larger Ca influx during dialysis with higher D[Ca] in patients suggestive of low bone turnover leads to vascular calcification and subsequent MI in DM.

## Introduction

Patients with end-stage renal disease have high cardiovascular (CV) morbidity and mortality rate compared with general population^1–3^. In addition to traditional risk factors, mineral and bone disorder has been reported to be strongly associated with high CV morbidity and mortality among dialysis population^4–11^.

Dialysate calcium concentration (D[Ca]) is one of the modifiable factors which play important roles in mineral and bone disorder^12–14^. Dialysis Outcomes and Practice Patterns Study showed that higher D[Ca] was associated with all-cause mortality^7^. However, other studies showed variable association of D[Ca] with mortality and CV morbidities^8,15,16^. These discrepancies raise the possibility that the effect of D[Ca] on CV morbidity is different in different patient population. Actually, several studies suggested that D[Ca] might have different impacts on vascular calcification or mineral and bone disorder depending on baseline patient characteristics^12,17^. However, association of D[Ca] and CV events was not evaluated in these studies due to short follow-up and small number of participants.

In this study, we investigated the association of D[Ca] and CV events and also investigated which patient characteristics affect the association using the database from the Japan Renal Data Registry (JRDR). In Japan, more than 90% of dialysis facilities use central dialysate supply system. Thus, there is little indication bias for D[Ca] for each individual patient. This gives us a unique opportunity for investigating the association between D[Ca] and CV events.

## Methods

### Study Design

This is a longitudinal study based on the JRDR database from 2008 to 2009. Details on the JRDR have been published previously^2^. Briefly, it is a database of all dialysis patients in Japan. At the end of every year, each dialysis unit voluntarily participates in data collection. The response rates were 99.0% and 98.5% in 2008 and 2009, respectively. The study protocol was approved by the Medicine Ethics Committee of the Japanese Society for Dialysis Therapy and the study was conducted in accordance with Helsinki Declaration. The waiver of consent for JRDR was also approved by the Ethics Committee. The complete de-identification has secured the privacy of the human subjects in our database, its secondary or unofficial use (i.e. any distribution to a third party, unauthorized replication or manipulation of database, and deviation from the proposal accepted by the Committee of Renal Data Registry) is strictly prohibited by the provision of agreements between the principle investigators and the Japanese Society for Dialysis Therapy, by which all rights regarding the database are reserved.

### Subjects

The inclusion criteria were prevalent hemodialysis patients who have been on hemodialysis for more than 1 year at the end of 2008, age 20–100 years old. The exclusion criteria were as follows: subjects who withdrew from dialysis, changed their treatment modality to peritoneal dialysis, or were transplanted, with missing data for D[Ca], history of CV events, or covariates used for adjustment, or apparent errors (ex. history of a CV event was present in 2008 but not in 2009).

### Exposure of interest and outcomes

**Figure 1:**
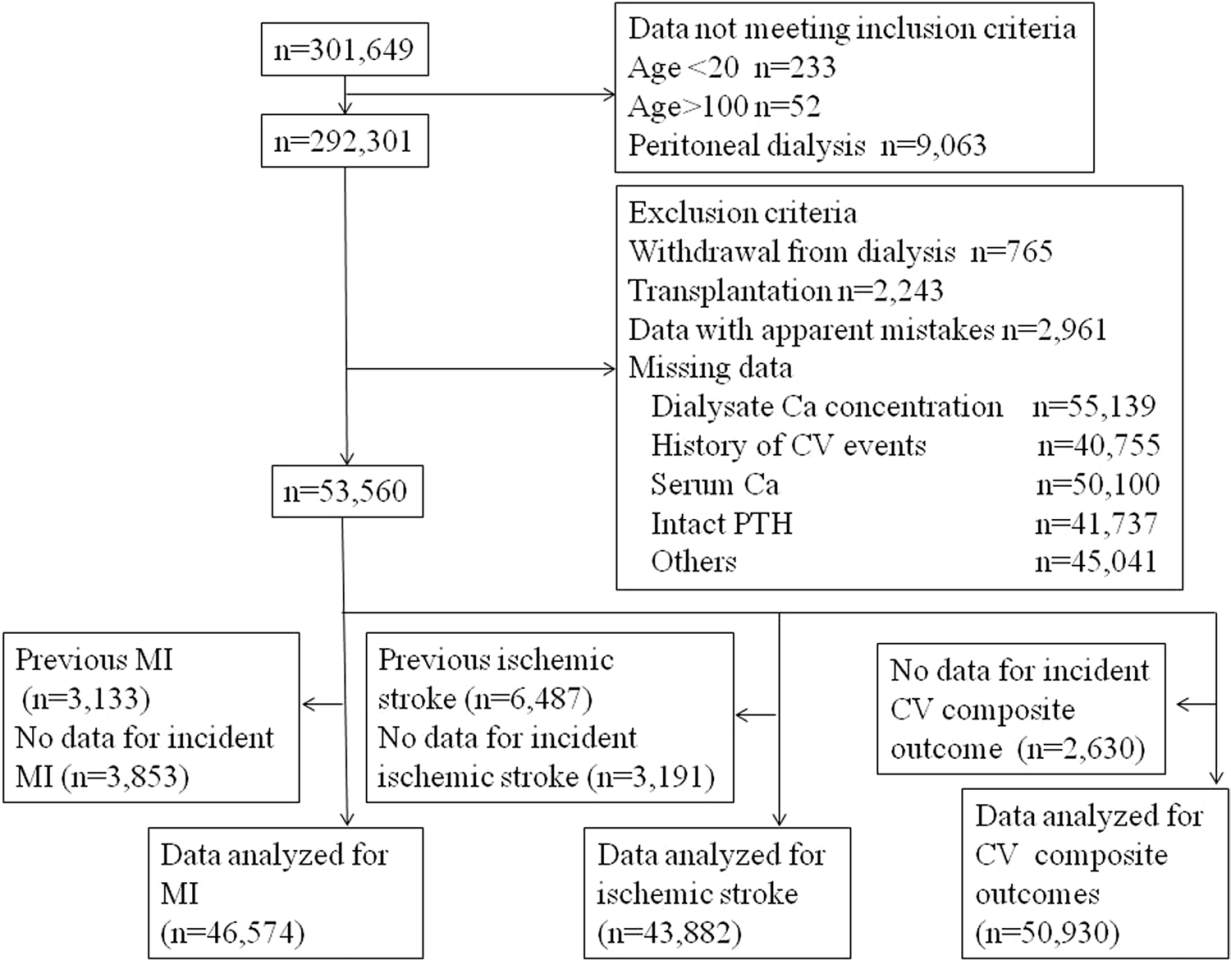
Flow of patients. As the Japan Renal Data Registry contains data on the histories of CV events at the end of each year, whether subjects had events during 2009 could not be determined for those with histories of CV events at the end of 2008. Thus, subjects with histories of MI, ischemic stroke at baseline were excluded from the analyses when the outcome variables were incident MI, or ischemic stroke, respectively. MI: myocardial infarction, CV: cardiovascular.

Exposure of interest was D[Ca] ≥3.0 mEq/L compared with 2.5 mEq/L in 2008, as D[Ca] of majority of commercially available dialysate at the time of the study in Japan was either 2.5 or 3.0 mEq/L. Outcome variables were the first episodes of CV events during the 1-year observation period in 2009. Subjects were considered to have incident events if they had no histories of these diseases in the 2008 database and did have histories of these diseases in the 2009 database. As the JRDR contains data on the histories of CV events at the end of each year, whether subjects had events during 2009 could not be determined for those with histories of CV events at the end of 2008. Thus, subjects with histories of MI or ischemic stroke at baseline were excluded from the analyses when the outcome variables were incident MI or ischemic stroke, respectively (Fig. 1).

### Definition

Serum albumin-corrected Ca was calculated as follows: corrected Ca (mg/dL) = serum calcium (mg/dL) + 0.8* [4-serum albumin (g/dL)] if serum albumin is less than 4 g/dL. Intradialytic change in serum Ca (ΔCa) was defined as post-dialysis serum Ca concentration – pre-dialysis serum Ca concentration (mg/dL). When calculating ΔCa, measured serum Ca levels were used, not albumin-corrected Ca.

### Statistical Analyses

Data were shown as n (%) for categorical variables, mean (SD) or median (interquartile range) for continuous variables with normal or non-normal distribution, respectively. The data were compared by chi-square test, independent sample t-test or Mann-Whitney-U test, as appropriate. The standardized difference (*d*) was calculated using the formula below.

$${\rm{d}}=\frac{M[Dca3]-M[Dca2.5]}{SD\,pooled}$$

$${\rm{SDpooled}}=\sqrt{\frac{(n[Dca3]-1)S{D}^{2}[DCa3]+(n[Dca2.5]-1)S{D}^{2}[DCa2.5]}{nDca3+nDCa2.5-2}}$$

M: mean, SD: standard deviation, n: number

Associations of D[Ca] and CV events were evaluated using multivariate logistic regression analyses adjusted for the following variables: age, sex, causes of end-stage renal disease, dialysis vintage, albumin, phosphate, quintiles of Kt/V, total cholesterol, C-reactive protein, ultrafiltration rate, body mass index, hemoglobin, and history of CV events other than the outcome variable. Corrected Ca and intact parathyroid hormone (iPTH) levels were not included in the initial model as they could be in the causal pathway between higher D[Ca] and CV events. Intact PTH levels were imputed from the 2007 database (last observation carried forward) as the data were not available in the 2008 database. Intact PTH were divided into four categories (≤60, 61–150, 151–240, >240 pg/ml) according to guidelines by Japanese Society for Dialysis therapy^18^. Logistic regression analyses were performed instead of survival analyses because the data indicated that the subjects had histories of each disease at the end of the year, but not when the events occurred during the year. Mediation analysis was performed using incident MI, D[Ca], and delta Ca as a dependent variable, an independent variable, and a mediator variable, respectively. Descriptive statistics and logistic regression analyses were performed using SPSS version 19.0 (SPSS Inc, Chicago, IL). Mediation analysis was performed using Stata MP version 15.0 (Stata Corp,College Station, Texas).

## Results

At the end of 2008, data were collected on 301,649 patients treated with dialysis in Japan. Among them, 292,301 patients met the inclusion criteria. After the exclusion of patients by the exclusion criteria, data from 53,560 patients were analyzed (Fig. 1). Excluded patients were older by 1.5 years and vintage was shorter by 1 year. The causes of end-stage renal diseases were similar and the proportion of patients using higher dialysate Ca concentration was also similar. The characteristics of the subjects included in the analyses were shown in Table 1. The proportion of patients using D[Ca] 2.5, 3.0, 3.3, and 3.5 mEq/L was 38.8%, 60.8%, 0.2%, and 0.2%, respectively. The patients using D[Ca] ≥3.0 mEq/L had significantly higher corrected Ca and  lower iPTH levels. There were statistically significant differences in age, causes of end-stage renal disease, dialysis vintage, body mass index, total cholesterol, C-reactive protein and KT/V among users of D[Ca] ≥3.0 mEq/L and 2.5 mEq/L, but the standardized difference was less than 10%. Serum phosphate was significantly lower among users of D[Ca] ≥3.0 mEq/L and the standardized difference was −9.94%. The number of incident events in 2009 among patients without prior events and incident rate (events/100 person-years) for MI, hemorrhagic stroke, ischemic stroke, limb amputation and CV composite outcomes (either MI, stroke, limb amputation or CV death) were 932 (1.8), 537 (1.0), 1,627 (3.2), 304 (0.6) and 5,095 (9.9), respectively. There were 186 death due to coronary artery disease. As the incidence of hemorrhagic stroke and limb amputation was low, subsequent subgroup analyses were performed for MI, ischemic stroke, and CV composite outcomes.

**Table 1:** Demographics.

		All (n = 53,560)	Dialysate Ca ≥3.0 mEq/L (n = 32,729)	Dialysate Ca 2.5 mEq/L (n = 20,831)	p	*d* (%)
Age		65.5 (12.4)	65.3 (12.5)	65.7 (12.3)	<0.001	−3.22
Male		32,668 (61.0)	19,906 (60.8)	12,762 (61.3)	0.31	
Causes of end-stage renal disease	Chronic glomerulo-nephritis	21,763 (40.6)	13,430 (41.0)	8,333 (40.1)	<0.001	
	Diabetes mellitus	17,070 (31.9)	10,208 (31.2)	6,862 (32.9)		
	Hypertension	3,958 (7.4)	2,516 (7.7)	1,442 (6.9)		
	Others	10,769 (20.1)	6,575 (20.1)	4,194 (20.1)		
Dialysis vintage (years)		9.1 (7.1)	9.2 (7.2)	9.0 (7.0)	<0.001	2.81
Ultrafiltration (L)		2.48 (1.16)	2.49 (1.17)	2.47 (1.14)	0.08	1.73
Phosphate (mg/dL)		5.28 (1.41)	5.22 (1.42)	5.36 (1.39)	<0.001	−9.94
Albumin-corrected calcium (mg/dL)		9.28 (0.78)	9.31 (0.78)	9.22 (0.79)	<0.001	11.48
Intact parathyroid hormone (pg/mL)		145 (71–251)	134 (64–235)	164 (84–273)	<0.001	−20.52*
Body mass index		21.2 (3.5)	21.1 (3.5)	21.2 (3.5)	0.03	−2.86
Albumin (g/dL)		3.7 (0.4)	3.7 (0.4)	3.7 (0.4)	0.30	0
Hemoglobin (g/dL)		10.5 (1.2)	10.5 (1.2)	10.5 (1.2)	0.99	0
Total cholesterol (mg/dL)		153 (36)	154 (35)	151 (37)	<0.001	8.38
C reactive protein (mg/dL)		0.11 (0.05–0.33)	0.10 (0.05–0.33)	0.11 (0.05–0.33)	<0.001	0*
Kt/V		1.43 (0.29)	1.43 (0.29)	1.42 (0.29)	0.01	3.45

Data shown as n (%), mean (SD), or median (interquartile range) as appropriate. P values were by chi-square test, independent sample t-test or Mann-Whitney-U test. *d*: standardized difference, *Standardized difference was calculated after log transformation.

**Figure 2:**
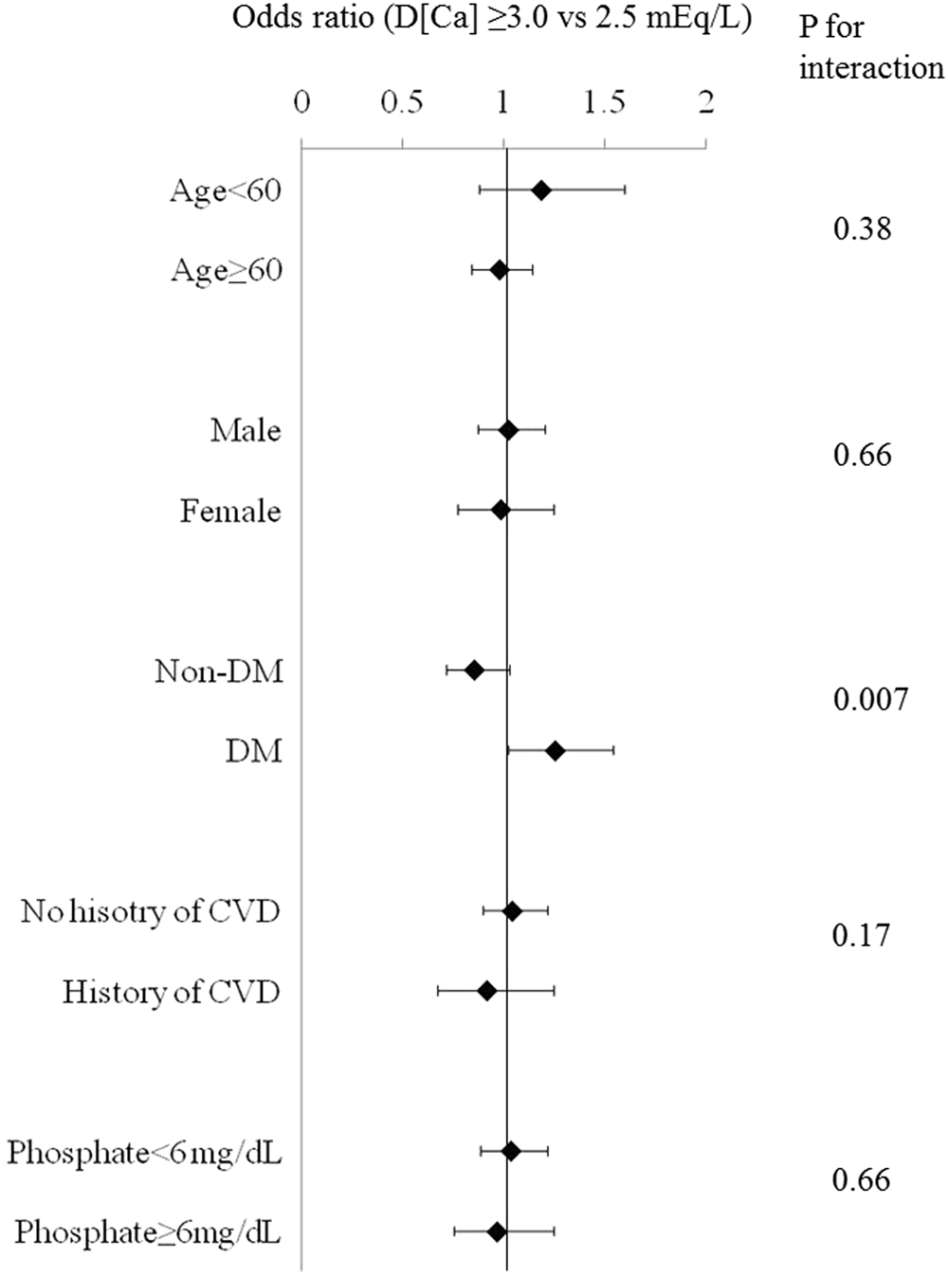
Association of dialysate calcium concentration and incident myocardial infarction in various subgroups. D[Ca]: dialysate calcium concentration, DM: diabetes mellitus, CVD: cardiovascular disease (either history of ischemic stroke, hemorrhagic stroke or limb amputation).

In the total cohort, there was no significant association between D[Ca] and CV events in both univariate and multivariate models (Table 2). Association of D[Ca] and CV events in different subgroups were examined. D[Ca] ≥3.0 mEq/L compared with 2.5 mEq/L was significantly associated with MI among patients with diabetes mellitus (DM) (Fig. 2). The ORs (D[Ca] ≥3.0 mEq/L compared with 2.5 mEq/L) for MI among diabetics and non-diabetics were 1.26 (1.03–1.55) and 0.86 (0.72–1.03), respectively (p for interaction = 0.007). There was no significant interaction in other subgroups (Fig. 2). Also, higher D[Ca] was not associated with ischemic stroke or CV composite outcomes in any subgroups (data not shown). Therefore, we focused on association of D[Ca] and MI, especially among diabetics. The demographics of patients stratified by diabetic status were shown in Supplementary Table 1. Albumin-corrected Ca and intact PTH were significantly lower among diabetics.

**Table 2:** Association of dialysate calcium concentration and cardiovascular events.

	Odds ratio (95% CI) (Dialysate Ca ≥3.0 mEq/L vs dialysate Ca 2.5mEq/L)	
	Univariate	Multivariate*
Myocardial infarction	0.99 (0.86–1.13)	1.01 (0.89–1.16)
Ischemic stroke	1.04 (0.94–1.15)	1.07 (0.96–1.19)
Cardiovascular composite outcome	0.97 (0.92–1.03)	1.00 (0.94–1.06)

^*^Data were adjusted for age, sex, causes of end-stage renal disease, dialysis vintage, albumin, phosphate, quintiles of Kt/V, total cholesterol, C - reactive protein, ultrafiltration rate, body mass index, hemoglobin, and history of myocardial infarction, hemorrhagic stroke, ischemic stroke, and limb amputation.

As the use of D[Ca] ≥3.0 mEq/L was associated with significantly higher corrected Ca and lower iPTH levels (Table 1), there was a possibility that association of D[Ca] and MI were through its effect on serum Ca or iPTH levels. Thus the model was further adjusted for corrected Ca and iPTH levels (Table 3). There was no change in the effect size. When the model was adjusted for ΔCa (post-dialysis serum Ca – pre-dialysis serum Ca level), the association of D[Ca] and MI was attenuated (Table 3). Mediation analysis was performed using incident MI, D[Ca], and delta Ca as a dependent variable, an independent variable, and a mediator variable, respectively. Data were adjusted for the same covariates as logistic regression analyses. Proportion of total effect mediated by delta Ca was 52%. These results suggest that D[Ca] ≥3.0 mEq/L was associated with MI through raising serum Ca rapidly during dialysis. Among non-diabetics using D[Ca] 2.5 mEq/L, non-diabetics using D[Ca] 3.0 mEq/L, diabetics using D[Ca] 2.5 mEq/L, and diabetics using D[Ca] ≥3.0 mEq/L, ΔCa (mean[SD]) was 0.02 (0.73), 1.00 (0.77), 0.04 (0.64), and 1.00 (0.71) mg/dL, respectively (p < 0.001 comparing D[Ca] ≥3.0 and 2.5 mEq/L among diabetics and non-diabetics, p = 0.02 comparing diabetics and non-diabetics among users of D[Ca] 2.5 mEq/L, and p = 0.59 comparing diabetics and non-diabetics among users of D[Ca] ≥3.0 mEq/L).

**Table 3:** Association of dialysate calcium concentration and myocardial infarction among diabetics and non-diabetics.

	Odds ratio (95% CI) (Dialysate Ca ≥3.0 mEq/L vs dialysate Ca 2.5 mEq/L)	
	DM	Non-DM
Model 1	1.26 (1.03–1.55)	0.86 (0.72–1.03)
Model 2	1.26 (1.03–1.55)	0.86 (0.72–1.03)
Model 3	1.30 (1.06–1.59)	0.87 (0.73–1.05)
Model 4	1.36 (1.10–1.68)	0.89 (0.74–1.08)
Model 5	1.16 (0.89–1.51)	0.85 (0.68–1.07)

Model 1: adjusted for age, sex, causes of end-stage renal disease, dialysis vintage, albumin, phosphate, quintiles of Kt/V, total cholesterol, C-reactive protein, ultrafiltration rate, body mass index, hemoglobin, and history of hemorrhagic stroke, ischemic stroke, and limb amputation. Model 2: adjusted for covariates in model 1 and pre-dialysis serum albumin-corrected calcium. Model 3: adjusted for covariates in model 2 and four categories of intact parathyroid hormone (≤60, 61–150, 151–240, >240 pg/mL). Model 4: adjusted for covariates in model 1 for 40,780 patients who have data for pre- and post-dialysis serum calcium. Model 5: adjusted for covariates in model 1 and change in serum calcium concentration during hemodialysis (post-dialysis serum calcium – pre-dialysis serum calcium). DM: diabetes mellitus.

**Figure 3:**
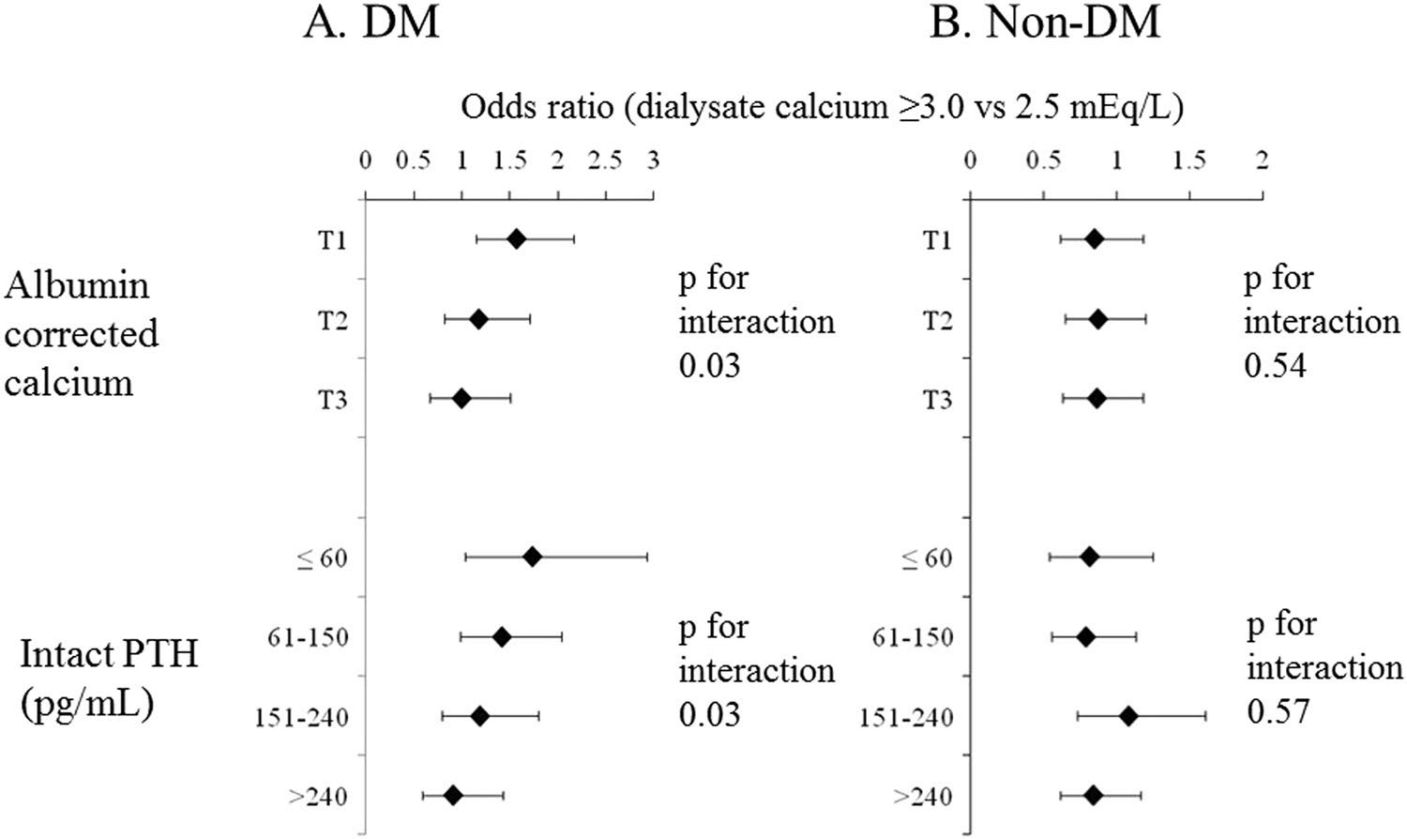
Association of dialysate calcium concentration and myocardial infarction, stratified by pre-dialysis serum albumin-corrected calcium and intact parathyroid hormone levels among diabetics and non-diabetics. DM: diabetes mellitus, PTH: parathyroid hormone, T1, T2, T3: first, second, and third tertiles of corrected calcium.

**Figure 4:**
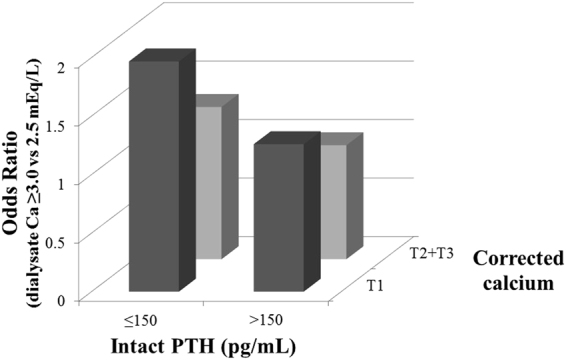
Association of dialysate calcium concentration and myocardial infarction in four categories of pre-dialysis serum albumin-corrected calcium and intact parathyroid hormone levels among diabetics. PTH: parathyroid hormone, T1, T2, T3: first, second, and third tertiles of corrected calcium.

Patient with low serum corrected Ca should have high ΔCa due to large concentration gradient of Ca between serum and dialysate. Also, patients with low iPTH might not be able to buffer Ca influx during hemodialysis due to low bone turnover. Association of D[Ca] and MI among diabetics and non-diabetics, stratified by corrected Ca and iPTH was examined and the results were shown in Fig. 3. Among diabetics, D[Ca] ≥3.0 mEq/L was significantly associated with MI in the first tertile of corrected Ca or iPTH <60 pg/mL (1.59 [1.16–2.17] and 1.75 [1.04–2.94], respectively). The effect size of D[Ca] ≥3.0 mEq/L was progressively reduced as corrected Ca and iPTH was higher (p for interaction 0.03 and 0.03, respectively). However, there was no effect modification by corrected Ca or iPTH among non-diabetics. Among diabetics, the effects of corrected Ca and iPTH on the association of D[Ca] and MI were additive and OR of D[Ca] ≥3.0 mEq/L was highest among patients with low corrected Ca and low iPTH levels (1.97 [1.25–3.11]) (p for interaction 0.014) (Fig. 4).

The association of ΔCa and MI was examined among users of D[Ca] ≥3.0 mEq/L (Table 4). Among all the users of D[Ca] ≥3.0 mEq/L, ΔCa was not significantly associated with MI, however, among diabetics, ΔCa was significantly associated with MI (1.28 [1.05–1.56]).

**Table 4:** Association of change in serum calcium concentration during dialysis and myocardial infarction among users of dialysate calcium concentration of ≥3.0 mEq/L.

	Odds ratio (95% CI) per 1 mg/dL change in serum calcium concentration during dialysis
All	1.11 (0.98–1.27)
Non-DM	0.99 (0.84–1.18)
DM	1.28 (1.05–1.56)

Adjusted for age, sex, causes of end-stage renal disease, dialysis vintage, serum albumin, phosphate, quintiles of Kt/V, total cholesterol, C reactive protein, ultrafiltration rate, body mass index, hemoglobin, history of hemorrhagic stroke, ischemic stroke and limb amputation, and four categories of intact parathyroid hormone. DM: diabetes mellitus.

## Discussion

In this study, the association of D[Ca] and CV events and its effect modifiers were examined. The major findings were as follows: higher D[Ca] was significantly associated with MI only among diabetics, especially with low corrected Ca or low iPTH levels, and this association was not through higher serum corrected Ca or lower iPTH levels but through rapid increase in serum Ca during hemodialysis with the use of higher D[Ca].

Previous studies showed variable association of D[Ca] and CV morbidity or mortality^7,8,15,16^. In our study, D[Ca] was not significantly associated with CV events in total cohort. These different associations of D[Ca] and CV events could be due to the different patient population. In fact, previous studies have showed that impact of D[Ca] may be different according to patient background. For example, when D[Ca] was changed from 3.0 to 2.75 mEq/L, iPTH levels increased among patients with low baseline iPTH, but iPTH levels did not change or slightly decreased among patients with high baseline iPTH^12^. Another study showed that progression of coronary calcification score was significantly larger among high D[Ca] users if baseline serum phosphate was ≥4.7 mg/dL but not if baseline serum phosphate was <4.7 mg/dL^17^. These studies unfortunately did not examine the association of higher D[Ca] and CV events as the number of patients included was small and also the follow-up was short. In our study, association of D[Ca] and different CV events in different subgroups were examined. D[Ca] was significantly associated with MI, but not other CV events, only among diabetics (Fig. 2). The difference in prevalence of DM and the incidence of MI might be an explanation for different association of D[Ca] and CV mortality in previous studies. Also, association of D[Ca] only with MI was similar to our previous study showing that Ca, phosphate, and iPTH levels were associated most with MI compared to other CV events^19^. Unlike a previous study^17^, phosphate level was not an effect modifier for the association of D[Ca] and CV events in our study (Fig. 2).

Despite the expectation that physicians would try to control corrected Ca and iPTH levels in target range recommended by the guideline^18^ irrespective of D[Ca], the users of higher D[Ca] had significantly higher corrected Ca and significantly lower iPTH levels (Table 1). Similar results were reported in previous studies^12,17,20^. However, the association of D[Ca] and MI among diabetics were not through the effect of D[Ca] on corrected Ca or iPTH, as the adjustment for these variables did not change the effect size (Table 3). Rather, adjustment for ΔCa attenuated the association of D[Ca] and MI among diabetics (Table 3), suggesting that association of D[Ca] and MI was through rapid increase in serum Ca during hemodialysis. In previous studies, association between D[Ca] and serum Ca, iPTH, arterial stiffness and vascular calcification was studied^12–14,17,21,22^, and it has been believed that these are the biological explanations for association of higher D[Ca] and CV events. However, at least, change in serum corrected Ca and iPTH with the use of higher D[Ca] does not seem to be the link between D[Ca] and MI from our results. To our knowledge, it has not been reported that rapid increase in serum Ca during HD is a possible link between D[Ca] and CV events.

Patients with low serum Ca should have larger ΔCa due to large concentration gradient of Ca between serum and dialysate. Also, patients with low iPTH levels are likely to have low bone turnover and may not buffer large Ca influx during dialysis. Thus, we performed stratified analyses by corrected Ca and iPTH. Higher D[Ca] was associated with MI especially in patients with low corrected Ca and iPTH among diabetics (Figs 3 and 4), suggesting that diabetic patients likely with low bone turnover could not buffer larger Ca influx with the use of higher D[Ca] and that might lead to vascular calcification and subsequent MI. Of note, the use of higher D[Ca] suppresses iPTH levels. Thus, the use of higher D[Ca] increases the number of patients with low iPTH, for whom, the use of higher D[Ca] was associated with increased incidence of MI. As this study is an observational study, low iPTH levels caused by higher D[Ca] and low iPTH with the use of lower D[Ca] were treated equally, which would underestimate the effect size. Actually, previous studies have shown that low iPTH induced by higher D[Ca] was associated with increased incidence of CV mortality^23^.

The significant effect modification by corrected Ca and iPTH and association of ΔCa and MI among higher D[Ca] users were only seen among diabetics (Fig. 3, Table 4). It is known that iPTH secretion is impaired in diabetics^24^ and it leads to low bone turnover. However, in our study, even among same strata of iPTH ≤60 pg/mL, D[Ca] was associated with MI only among diabetics. It has been reported that function and differentiation of osteoblasts and ostelclasts are impaired and these lead to low bone turnover in DM^25–27^. For example, advanced glycation end products (AGEs) act through receptor for AGEs on osteoblasts and inhibit osterix and runt-related transcription factor 2, which are necessary for osteoblast differentiation^25^ and induce transforming growth factor-β, which inhibits osteoblast differentiation^26^. AGEs also inhibit receptor activator of nuclear factor-κB ligand, which are necessary for osteoclast differentiation^27^. Our results further reinforce the notion that DM itself is the cause of low bone turnover, independent of iPTH in chronic kidney disease.

Strength of our study is as follows: this is a large cohort of hemodialysis patients and associations of D[Ca] with hard outcomes were examined. In Japan, more than 90% dialysis facility use central dialysate supply system. This means that the choice of dialysate Ca concentration is center-based and not based on patient characteristics such as serum Ca or iPTH levels and there is little indication bias for D[Ca] for each individual patient. Thus, JRDR is a unique population for investigating the association between D[Ca] and CV events. Also, to our knowledge, this is the first study showing that rapid increase in serum Ca during hemodialysis probably from large concentration gradient of Ca between serum and dialysate and also from low buffering capacity of bone is a possible mechanism to explain association of higher D[Ca] and CV events.

There are several limitations in our study. As this was an observational study, there was a possibility of residual confounders. Data for the use of vitamin D, cinacalcet and phosphate binders were not available in the database. It is known that more vitamin D is used among patients dialyzed by lower D[Ca]^20^. However, the association between dialysate Ca concentration and MI was mediated through intradialytic change in serum Ca concentration and it is unlikely that vitamin D, cinacalcet, and phosphate binders have direct impact on intradialytic change in serum Ca concentration. Also, although we do not have exact data, cinacalcet was introduced to Japanese market in 2008, and the number of patients on cinacalcet was presumed to be small at the time of the study. Also, as we could not determine whether patients with history of CV events at baseline had recurrent events during the observation period, the incidence of CV events should have been underestimated. Large number of subjects was excluded from analyses due to missing data and demographics of excluded and included subjects were different. However, it is unlikely that data were systematically missed in relation to exposure (i.e. D[Ca]) and it is unlikely to affect the results. The data for D[Ca] and post-dialysis serum calcium concentration were only available in 2008 in JRDR database. As the use of lower D[Ca] increased over time since then in Japan, we limited the observation period to one year to examine the effect of D[Ca] and delta Ca on outcomes.

In conclusion, we showed that higher D[Ca] was associated with incident MI among diabetics, especially with low serum Ca and iPTH levels. The attenuation of effect size by adjustment for ΔCa and stratified analyses suggest that larger Ca influx with the use of higher D[Ca] in patients likely with low bone turnover lead to vascular calcification and subsequent MI. The results suggest that D[Ca] ≥3.0 mEq/L should be avoided in diabetic patients with low serum Ca or iPTH levels.

## Electronic supplementary material

Suuplementary Table 1
