# Author Correction: Higher dialysate calcium concentration is associated with incident myocardial infarction among diabetic patients with low bone turnover: a longitudinal study

**DOI:** 10.1038/s41598-018-31447-w

**Published:** 2018-08-30

**Authors:** Miho Tagawa, Takayuki Hamano, Shinichi Sueta, Satoshi Ogata, Yoshihiko Saito

**Affiliations:** 1 Department of Nephrology, Nara Medical University, Kashihara, Japan; 2 Committee of Renal Data Registry, Japanese Society for Dialysis Therapy, Tokyo, Japan; 3 Center for iPS Cell Research and Application, Kyoto University, Kyoto, Japan; 4 First Department of Internal Medicine, Nara Medical University, Kashihara, Japan

Correction to: *Scientific Reports* 10.1038/s41598-018-28422-w, published online 03 July 2018

This Article contains an error in the Acknowledgements section.

“The standard analysis file (JRDR-09004) was used for this study.”

should read:

“The standard analysis file (SAF-2016-010) was used for this study.”

