# Supplementary material for: Higher dialysate calcium concentration is associated with incident myocardial infarction among diabetic patients with low bone turnover: a longitudinal study
Source: Sci Rep. 2018 Jul 3;8:10060. doi: 10.1038/s41598-018-28422-w (PMC6030065; doi:10.1038/s41598-018-28422-w)
Supplement: Supplementary file 1 — Suuplementary Table 1 [file 41598_2018_28422_MOESM1_ESM.docx]

Higher dialysate calcium concentration is associated with incident myocardial infarction among diabetic patients with low bone turnover: a longitudinal study

Miho Tagawa, PhD, Takayuki Hamano, PhD, Shinichi Sueta, MD, Satoshi Ogata, PhD, Yoshihiko Saito, PhD

Supplementary Table 1. Demographics stratified by diabetic status

|  | | Non-diabetics  (n=36,490) | Diabetics  (n=17,070) | p | *d* (%) |
| --- | --- | --- | --- | --- | --- |
| Age | | 65.1 (13.1) | 66.4 (10.9) | <0.001 | 10.5 |
| Male | | 21,288 (58.3) | 11,380 (66.7) |  |  |
| Causes of end-stage renal disease | Chronic glomerulo-nephritis | 21,763 (59.7) | 0 | <0.001 |  |
|  | Diabetes mellitus | 0 | 17,070 (100) |  |  |
|  | Hypertension | 3,958 (10.8) | 0 |  |  |
|  | Others | 10,769 (29.5) | 0 |  |  |
| Dialysis vintage (years) | | 10.6 (7.8) | 5.9 (3.7) | <0.001 | -69.0 |
| Ultrafiltration (L) | | 2.4 (1.2) | 2.6 (1.2) | <0.001 | 16.2 |
| Phosphate (mg/dL) | | 5.31 (1.40) | 5.20 (1.43) | <0.001 | -7.7 |
| Albumin-corrected calcium  (mg/dL) | | 9.33 (0.79) | 9.16 (0.76) | <0.001 | -22.2 |
| Intact parathyroid hormone  (pg/mL) | | 155 (76-271) | 128 (63-212) | <0.001 | -19.6* |
| Body mass index | | 20.8 (3.3) | 22.0 (3.8) | <0.001 | 35.4 |
| Albumin (g/dL) | | 3.7 (0.4) | 3.7 (0.4) | <0.001 | -8.3 |
| Hemoglobin (g/dL) | | 10.5 (1.2) | 10.4 (1.2) | 0.005 | -2.6 |
| Total cholesterol (mg/dL) | | 155 (36) | 150 (36) | <0.001 | -12.1 |
| C reactive protein (mg/dL) | | 0.10  (0.05-0.31) | 0.12  (0.05-0.37) | <0.001 | 8.3* |
| Kt/V | | 1.46 (0.29) | 1.35 (0.27) | <0.001 | -40.4 |

Data shown as n (%), mean (SD), or median (interquartile range) as appropriate. P values were by chi-square test, independent sample t-test or Man-Whitney U test. *d*: standardized difference, *Standardized difference was calculated after log transformation.
